# Metabolomic and Pharmacologic Insights of Aerial and Underground Parts of *Glycyrrhiza uralensis* Fisch. ex DC. for Maximum Utilization of Medicinal Resources

**DOI:** 10.3389/fphar.2021.658670

**Published:** 2021-06-01

**Authors:** Liang Jiang, Waheed Akram, Biaobiao Luo, Sheng Hu, Mohammad Omar Faruque, Shakeel Ahmad, Nasim Ahmad Yasin, Waheed Ullah Khan, Aqeel Ahmad, Alexander N. Shikov, Jian Chen, Xuebo Hu

**Affiliations:** ^1^ Department of Head and Neck Surgery, Hubei Cancer Hospital, Tongji Medical College, Huazhong University of Science and Technology, Wuhan, China; ^2^ Laboratory of Drug Discovery and Molecular Engineering, Department of Medicinal Plants, College of Plant Science and Technology, Huazhong Agricultural University, Wuhan, China; ^3^ National and Local Joint Engineering Research Center for Medicinal Plant Breeding and Cultivation, Wuhan, China; ^4^ Hubei Provincial Engineering Research Center for Medicinal Plants, Wuhan, China; ^5^ Ethnobotany and Pharmacognosy Lab, Department of Botany, University of Chittagong, Chittagong, Bangladesh; ^6^ ROII Office, University of the Punjab, Lahore, Pakistan; ^7^ Saint-Petersburg State Chemical Pharmaceutical University, Saint-Petersburg, Russia

**Keywords:** licorice, inflammation, henicosane, pro-inflammatory cytokines, metabolomics

## Abstract

The roots of *Glycyrrhiza* spp. have been utilized in Traditional Chinese medicine (TCM) for thousands of years. Non-traditional (aerial) parts constitute a large portion of the biomass of Glycyrrhiza plants and are mostly discarded after harvesting the roots and rhizomes. Through comparative phytochemical and anti-inflammatory activity analyses, this study explored the potential benefits of the aerial parts of *Glycyrrhiza uralensis* Fisch. ex DC. as medicinal materials. First, a combined approach based on GC/MS and UHPLC-ESI-QTof MS analysis was adopted for the identification and quantitative examination of medicinally important compounds from *G. uralensis*. Additionally, a bioassay-guided fractioning of ethanolic extracts of *G. uralensis* leaf material was performed and its anti-inflammatory activity was tested. The aerial portion of *G. uralensis* was rich in medicinally important compounds. Two compounds (henicosane-1 and decahydroisoquinoline-2) were found to exert a significant anti-inflammatory effect, inhibiting the release of pro-inflammatory mediators (NO and PGE2) and cytokines (IL-1β, IL6, and TNF-α), without exerting cytotoxic effects. Moreover, both compounds down-regulated iNOS and COX-2 mRNA expression. These results suggest that non-traditional parts of *G. uralensis* are suitable sources of bioactive metabolites that can be explored for medicinal purposes.

## Introduction

*Glycyrrhiza uralensis* Fisch. ex DC. as well as *Glycyrrhiza glabra* L. (Fabaceae), commonly known as licorice, are traditional plants recognized through ages for their multiple health benefits and medicinal uses. Particularly, *G. uralensis* is mentioned in the pharmacopoeia of China, Russia, and other countries (Wang et al., 2020). “Licorice” is obtained from the underground parts of *G. uralensis* and related species. Different compounds including triterpenoid saponins, flavanones, chalcones, and coumarins have been isolated from the roots of *Glycyrrhiza* spp. (Wang et al., 2020). *Glycyrrhiza* spp. are widely cultivated, since these contain most of the bioactive compounds that are responsible for their medicinal and culinary attributes as a flavoring agent and spice (Bell et al., 2011; Dong et al., 2014). Currently licorice is used at different stages of processing grains and oil products, meat products, beverages, candies, jellies, dried fruits, seeds, and soy sauce etc. (Montoro et al., 2011). The roots of this plant are used to treat influenza, coughs, and liver damage in traditional medicinal formulations (Zarubaev et al., 2016).

**FIGURE 1:**
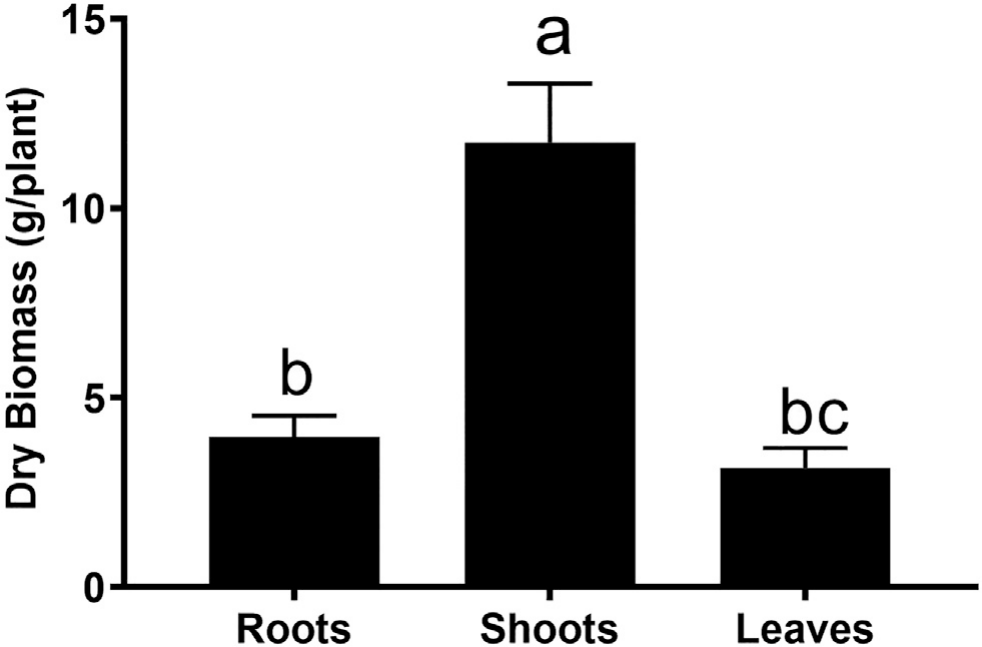
Comparative dry biomass of different parts of *G. uralensis*. Small letters represent level of significance among different treatments as inferred by DNMRT at *p* = 0.05.

Previous studies have shown that the extracts of the roots of *G. uralensis* contain antioxidant, anti-inflammatory, antiviral, cytotoxic, antidiabetic, inhibitors of angiotensin-converting enzyme 2 and transmembrane protease, serine 2, skin-whitening, hepatoprotective, and cholinergic properties (Ahn et al., 2010; Gou et al., 2020; Isbrucker and Burdock 2006; Wu et al., 2020). However, the aerial portion of this plant is of lesser importance to cultivators and usually constitutes an agro-industrial waste after the harvest of the roots or rhizomes that corresponds to merely one fourth of the whole biomass of the plant (Figure 1). It is worth mentioning that the aerial parts of *G. uralensis* also contains liquiritin and some other medicinally important compounds (Table 1). While previous studies are mostly limited to some major compounds identified from the roots such as glycyrrhizin, liquiritin, liquiritigenin, and isoliquiritigenin (Ji et al., 2016), the information related to the medicinal importance of many other compounds present in the foliar portion of this plant is scarce. Therefore, in this study we examined the leaves of *G. uralensis* for the presence of anti-inflammatory compounds. To the best of our knowledge, we described for the first time the presence of two bioactive compounds in the leaves of this plant, and reported the effect of the isolated compounds on the production of important pro-inflammatory mediators.

**GRAPHICAL ABSTRACT:**
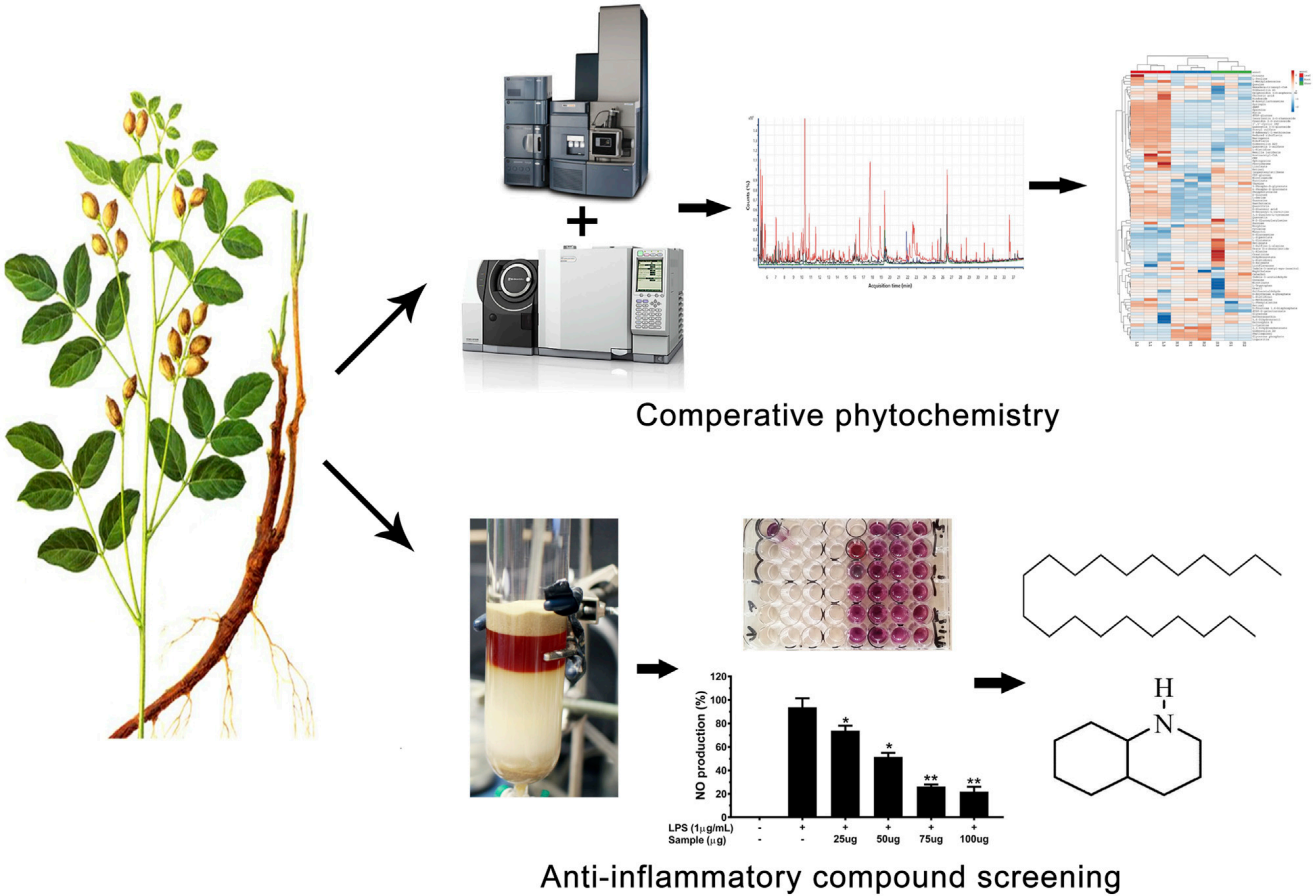


**TABLE 1:** Details of different compounds detected in leaf material of G. uralensis by performing GC/MS and LC/MS analysis.

No	RT	Compound	Identification method	Measured mass (*m/z*)	(*m/z*) Fragments	Formula	Mass
1	33.05	1,4-Piperazinediethanol	GC/MS		71, 83, 111	C_22_H_30_N_2_O_4_	368
2	26.14	1,3-Hydroxydocosanoic acid	UHPLC/MS	217 [M + H]+	179, 299	C_12_H_24_O_3_	216
3	54.07	2(1H)-Naphthalenone	GC/MS		109, 123, 177, 207	C_12_H_20_O	180
4	18.5	2-Propenoic acid	GC/MS		45, 55, 72	C_9_H_8_O_3_	164
5	21.3	3,5-Di-t-butylphenol	GC/MS		57, 163, 191, 207	C_14_H_22_O	206
6	0.69	3-Phosphoglycerate	UHPLC/MS	186 [M + H]+	118, 381, 465	C_3_H_7_O_7_P	186
7	0.60	4-Aminobutanoate	UHPLC/MS	103 [M + H]+	125, 203, 249	C_4_H_8_NO_2_	102
8	12.4	4-Methoxychalcone	UHPLC/MS	239 [M + H]+	287, 595, 596	C_16_H_14_O2	238
9	29.28	6-Phosphogluconic acid	UHPLC/MS	277 [M + H]+	277, 407, 553	C_6_H_13_O_10_P	276
10	13.07	Acenocoumarol	UHPLC/MS	354 [M + H]+	299, 371, 372	C_19_H_15_NO_6_	353
11	2.36	Allopurinol	UHPLC/MS	135[M-H]−	104,110,126,129	C_5_H_4_N_4_O	136
12	31.61	Galactose	UHPLC/MS	195 [M-H]−	423, 493	C_7_H_14_O_6_	194
13	35.50	Alpha-D-glucopyranoside	UHPLC/MS	195 [M + H]+	283, 305, 349, 415	C_7_H_14_O_6_	194
14	27.27	ATP	UHPLC/MS	505 [M-H]−	339, 679, 822	C_10_H_16_N_5_O_13_P_3_	504
15	52.5	Bromoacetic acid	GC/MS		44, 69, 83, 111	C_20_H_39_BrO_2_	390
16	33.69	Canrenone	UHPLC/MS	341 [M + H]+	283, 305, 360, 505	C_22_H_28_O_3_	340
17	17.04	Chelidonine	UHPLC/MS	354 [M + H]+	271, 315, 355, 356	C_20_H_19_NO_5_	353
18	14.45	Cholic acid	UHPLC/MS	407[M-H]−	283, 355, 356	C_24_H_40_O_5_	408
19	21.9	Cyclohexanol	GC/MS		40, 69, 81, 109	C_6_H_12_O	100
20	17.50	Cyclopentadecanone	GC/MS		40, 69, 83	C_15_H_29_NO	239
21	11.25	Decahydroisoquinoline	GC/MS		30, 44, 96, 138	C_9_H_17_N	139
22	27.32	Fructose 1,6-bisphosphate	UHPLC/MS	341 [M + H]+	153, 449, 734	C_6_H_14_O_12_P_2_	340
23	5.17	Glucose 6-phosphate	UHPLC/MS	259 [M-H]−	78, 96, 168	C_6_H_13_O_9_P	260
24	13.25	d-Glutamic acid	GC/MS		84, 102	C_5_H_9_NO_4_	147
25	21.25	Dihydrobenzimidazol	GC/MS		249, 305, 361	C_19_H_34_N_2_OSi_2_	362
26	33.95	Dihydroquercetin	UHPLC/MS	303[M-H]-	283, 305, 349, 409	C_15_H_12_O_7_	304
27	16.76	Dihydroxy benzoate	GC/MS		44, 71, 141	C_9_H_10_O_4_	154
28	52.78	Docosanoic acid	GC/MS		73, 221, 281, 355	C_44_H_88_O_2_	684
29	29.69	Ergosterol	UHPLC/MS	397 [M + H]+	285, 341	C₂₈H₄₄O	396
30	34.89	Eriodictyol-7-O-glucoside	UHPLC/MS	451 [M + H]+	305, 349, 411, 451	C_21_H_22_O_11_	450
31	54.4	Fluoropropionate	GC/MS		57, 71, 97, 111	C_29_H_53_F_5_O_2_	528
32	35.41	Gibberellin A1	UHPLC/MS	349 [M + H]+	124, 261, 305, 423	C_19_H_24_O_6_	348
33	24.57	Gibberellin A8	UHPLC/MS	365 [M + H]+	255, 309, 399	C_19_H_24_O_7_	364
34	30.38	Ginkgolide B	UHPLC/MS	423 [M-H]−	369, 425, 426	C_20_H_24_O_10_	424
35	12.17	Henicosane	GC/MS		57, 71, 40	C_21_H_44_	296
36	63.7	Heptacosyl acetate	GC/MS		43, 69, 97, 111	C_29_H_58_O_2_	438
37	10.58	Herniarin	GC/MS		133, 148, 176	C_10_H_8_O_3_	176
38	18.25	Hexacosanoic acid	GC/MS		43, 57, 60, 73	C_26_H_52_O_2_	396
39	11.25	Hexadecane	GC/MS		79, 59, 43	C_16_H_34_	226
40	57.23	Hexadecanoic acid	GC/MS		43, 74, 87, 143	C_17_H_34_O_2_	270
41	37.60	Isoorientin	UHPLC/MS	447 [M-H]−	216, 339, 401	C_21_H_20_O_11_	448
42	36.61	Isorhamnetin-3-O-glucoside	UHPLC/MS	479 [M + H]+	439, 479, 480	C_22_H_22_O_12_	478
43	13.37	Alanine	UHPLC/MS	90 [M-H]-	89, 113, 139	C_3_H_7_NO_2_	89
44	38.27	Histidinol	UHPLC/MS	141 [M + H]+	69, 90, 165, 291	C_6_H_11_N_3_O	141
45	12.28	Proline	UHPLC/MS	115 [M + H]+	90, 115, 139	C_5_H_9_O_2_	115
46	3.18	Tryptophan	UHPLC/MS	205 [M + H]+	146, 170, 205	C_11_H_12_N_2_O_5_	204
47	38.57	Lycorine	UHPLC/MS	288 [M + H]+	288, 304	C_16_H_17_NO_4_	287
48	38.7	Mannitol	UHPLC/MS	183 [M + H]+	113, 128, 158, 182	C_6_H_14_O_6_	182
49	14.87	Morphine	UHPLC/MS	286 [M + H]+	129, 285, 287	C_17_H_19_NO_3_	285
50	38.62	Naphthalene	UHPLC/MS	128 [M + H]+	113, 141, 158, 169	C_10_H_8_	128
51	16.21	Naringenin-O-GluA	UHPLC/MS	447 [M-H]−	331, 417, 219	C_21_H_20_O_11_	448
52	56.97	n-Docosanol	GC/MS		83, 111, 152, 217	C_22_H_46_O	326
53	57.85	Octadecadienoic acid	GC/MS		55, 67, 82, 110	C_19_H_34_O_2_	294
54	50.2	Octadecenal	GC/MS		44, 73, 221	C_18_H_34_O	266
55	53.4	Octatriacontadiene	GC/MS		55, 69, 83, 111	C_38_H_74_	530
56	19.9	Ouabain	UHPLC/MS	585 [M + H]+	142, 170, 337	C_29_H_44_O_12_	584
57	2.03	Pantothenic acid	UHPLC/MS	220 [M + H]+	87, 103, 123	C_9_H_17_NO_5_	219
58	21.63	p-Cresol	GC/MS		205, 220	C_15_H_24_O	220
59	61.71	Pentafluoropropanoate	GC/MS		85, 208, 447	C_22_H_39_F_5_O_2_	430
60	53.6	Phthalic acid	GC/MS		57, 71, 149	C_23_H_36_O_4_	222
61	68.7	Phytol	GC/MS		44, 71, 81	C_20_H_40_O	296
62	11.38	Quercetin	UHPLC/MS	303 [M + H]+	128, 465, 611	C_15_H_10_O_7_	302
63	29.01	Quercetin 3-sulfate	UHPLC/MS	383 [M + H]+	139, 259, 327	C_15_H_10_O_10_S	382
64	23.45	Quinidine	UHPLC/MS	325 [M + H]+	142, 257, 415	C_20_H_24_N_2_O_2_	324
65	11.41	Rutin	UHPLC/MS	611 [M + H]+	303, 304, 611, 612	C_27_H_30_O_16_	610
66	28.92	S-Adenosyl-l-methionine	UHPLC/MS	399 [M + H]+	339, 383, 399	C_15_H_22_N_6_O_5_S	398
67	26.11	Sinapic acid	UHPLC/MS	225 [M + H]+	299, 355, 357, 358	C_11_H_12_O_5_	324
68	13.57	Stearic acid	GC/MS		43, 60, 73, 129	C_18_H_36_O_2_	284
69	18.24	Stigmasterol	GC/MS		55, 69, 83, 105, 133	C_29_H_48_O	412
70	27.27	Usnic acid	UHPLC/MS	343 [M-H]−	116, 399, 679		
71	32.17	β-D-glucopyranoside	UHPLC/MS	195 [M + H]+	283, 3.5	C_7_H_14_O_6_	194
72	33.68	Isoorientin 2-O-rhamnoside	UHPLC/MS	595 [M + H]+	305, 431, 773	C_27_H_30_O_16_	594
73	34.43	3′,5′-Cyclic AMP	UHPLC/MS	268 [M + H]+	284, 285, 286	C_10_H_13_N_5_O_4_	267
74	30.45	Queuine	UHPLC/MS	278 [M + H]+	227, 305	C_20_H_24_N_2_O_2_	324
76	10.77	Cyanidin 3-O-rutinoside	UHPLC/MS	596 [M + H]+	213, 287, 433	C_21_H_21_O_10_	595
77	35.00	Okadaic acid	UHPLC/MS	805 [M + H]+	681, 749, 769	C_44_H_68_O_13_	804
78	35.76	Acetylgdigitoxin	UHPLC/MS	851 [M + H]+	235, 385, 429	C_43_H_66_O_14_	850
79	33.14	Antheraxanthin	UHPLC/MS	585 [M + H]+	504, 567, 584	C_40_H_65_O_3_	584

Compounds that were identified by both techniques, identification details are provided of only UHPLC/QToF-MS analysis.

## Materials and Methods

### Chemicals and Reagents

All solvents and standards used for GC/MS and UHPLC ESI-QTof MS analysis were of chromatography grade and obtained from Sigma-Aldrich (St. Louis, MO, United States). Dulbecco’s modified Eagle’s medium (DMEM), penicillin, streptomycin and fetal bovine serum (FBS) were purchased from Thermo Scientific (Logan, UT, United States). Griess reagent, acetylsalicylic acid (aspirin), dimethylsulfoxide (DMSO), lipopolysaccharide (LPS), pyridine, ribitol were obtained from Sigma-Aldrich (St. Louis, MO, United States). MOX and MSTFA mixtures were purchased from Thermo Fisher (TX, United States). 13C-ribitol was obtained from Omicron Biochemicals Inc, (IN, United States). ELISA kits were obtained from Bio-Rad (CA, United States) and Sigma-Aldrich (St. Louis, MO, United States). The RAW 264.7 cell lines at sixth passage were used that were originally purchased from Sigma-Aldrich (St. Louis, MO, United States). Deionized water (Milli-Q) was used in the whole study (Millipore, Billerica, MA, United States).

### Comparative Metabolomics of the Roots and Aerial Parts of *G. uralensis*

#### Plant Material and Preparation of the Extracts

Different parts of *G. uralensis* were obtained from Qinghaihu Pharmaceutical, Co. Ltd. (Qinghai, China). Prof. Dr Xuebo Hu, from College of Plant Sciences and Technology, Huazhong Agricultural University, China, verified the identity of plant material (The specimens were kept at Institute for Medicinal Plants, Huazhong Agricultural University with voucher number 2017-Gu-0001, 2017-Gu-0002 and 2017-Gu-0003 for the roots, stems and shoots). In order to obtain an extract, the air-dried powdered (500 mg) material of roots and shoots of *G. uralensis* was macerated separately in 25 ml of MeOH/CHCl_3_/H_2_O (2.5:1:0.5, v/v) solution overnight under continuous stirring (Weckwerth et al., 2004). The whole process was performed twice. The material was filtered through a Whatman No. 1 filter paper. The solvent was evaporated using rotary evaporator under low-pressure to obtain a semi-solid consistency.

#### Extract Derivatization and GC/MS Analysis

Gas chromatography coupled with mass spectrometry (GC/MS) analysis was performed as described by Weckwerth et al. (2004) with some modifications. Here, 13C-ribitol (0.02 μg/μL) was used as an internal standard. Dried samples were derivatized using standard MOX and MSTFA mixtures as described by Mari et al. (2013). The clear supernatant was obtained after centrifugation and poured into clean GC-vials for analysis. The sample (1 µL) was injected in a Shimadzu GC/MS-QP2010 SE (Shimadzu, Japan) instrument at the constant temperature of 230°C in splitless mode. Chromatographic separation was performed using HP-5MS capillary column (30 m × 0.25 mm × 0.25 µm) and helium as carrier gas at a constant flow rate of 1.0 ml/min. The GC/MS temperature gradient used during analysis was same as adopted by Mari et al. (2013). Mass analyzer was set at full scan mode (40–800 m/z) and the ion source temperature was maintained at 250°C, with EI ionization at 70 eV.

#### UHPLC ESI-QTof MS analysis

**FIGURE 2:**
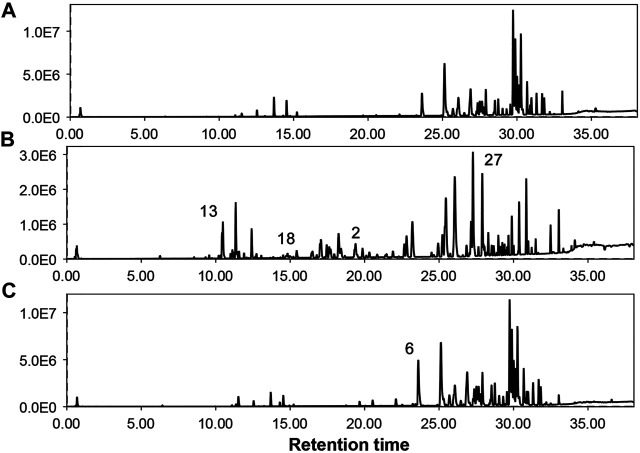
Total ion chromatograms of different parts of *G. uralensis* obtained from UHPLC-QTOF/MS analysis. **(A)** = Shoots, **(B)** = Leaves, **(C)** = Roots. Numbers over peaks represents different compounds as mentioned in Table 2.

Previously prepared plant extracts were dissolved in pure methanol following sonication for 5 min. The obtained solution was centrifuged, and the supernatant was passed through cellulose filters (0.2 µm pore size). Afterward, the sample (0.2 μL) was injected in an ultra-performance liquid chromatography coupled with electrospray ionization-quadrupole time-of-flight mass spectrometry (UHPLC-ESI-QTof MS/MS) instrument. The chromatographic separation (Figure 2) was performed on a Waters ACQUITY UHPLC I-class system (Waters Corporation, Dublin, Ireland) fitted with a Waters ACQUITY UHPLC BEH C18 column. The mobile phases were deionized H_2_O containing 0.1% of formic acid (A) and MeOH containing 0.1% of formic acid (B) at constant flow rate of 0.7 ml/min. The gradient was set as follows: 5% B at 0 min, linearly increasing from 5 to 10% B within 5 min, from 10 to 100% B within 22.5 min, and held at 100% B for 2.5 min. The chromatographic system was coupled with Waters Xevo QTof-MS *system* via an *electrospray ionization* (*ESI*) interface operating in full scan mode. The ESI source and MS parameters were set as adopted by (Muema et al., 2017).

#### Compound Identification and Data Analysis

MzMine version 2.30 (mzmine.github.io) was used for both qualitative and quantitative analysis of both GC/MS and LC/MS data. The alignment was carried out as a function of retention time, using a tolerance window of 0.2 min and 10 ppm mass accuracy (Molina-Calle et al., 2017). Metabolites were identified by comparing mass spectra with spectral libraries (NIST and Wiley), online database MassBank (http://www.massbank.jp/) and previously published literature (Zhang and Ye 2009; Siracusa et al., 2011; Farag et al., 2012; Ammar et al., 2017; Yu et al., 2017; Hefny Gad et al., 2018). Metabolites were identified with a spectral match factor higher than 800. The resulting data matrix based on the relative abundance of metabolites of different parts of *G. uralensis* was exported into the online tool ClustVis (https://biit.cs.ut.ee/clustvis/) to create heat maps and principal component analysis (PCA) plots. All samples were analyzed three times and mean data was used to perform statistical analysis. Furthermore, comparative quantifications of different medicinally important compounds were performed for their prevalence in aerial and below ground parts of *G. uralensis*.

### Isolation and Identification of Anti-inflammatory Compound/s from the Leaves of *G. uralensis*

#### Compound Extraction and Isolation

An overview of the purification process of compounds is shown in Supplementary Figure S1. Briefly, dried leaves of *G. uralensis* were ground into a fine powder prior to use. Leaf powder (∼1 kg) was first extracted with ∼20 L of EtOH at ∼ 77°C for 5 h. Afterward, the solvent was removed by rotary evaporator and lyophilized to obtain a dry material. This dried material was further extracted using an EtOH-H_2_O (v/v) based solvent system and a stepwise elution method with increasing EtOH concentration (from 20 to 100%) to yield five fractions (F1-F5). Subsequently, the selected bioactive sub-fractions, eluted at 60% EtOH (F 4.6) and 70% EtOH (F 4.7), were processed with column chromatography on silica gel to yield five sub-fractions (A–E). Parent fractions were separated into sub-fractions based on thin layer chromatography (TLC) to obtain pure compounds. Sub-fraction 4.6°C was passed through a silica gel column to obtain four sub-fractions, 4.6Ca–4.6Cd. Then, sub-fraction 4.6 Cb was purified on silica gel eluted with EtOH-H_2_O (3:1 v/v) to obtain compound 1 (6.8 mg). Similarly, compound 2 (826.5 mg) was purified using EtOH-H_2_O (4:1 v/v) from sub-fraction 4.7Bc. Purified compounds were identified by comparing spectral data with previously published data and authentic pure reference compounds.

#### Cell Line and Cell Culture

The cell line RAW 264.7 was maintained in DMEM medium supplemented with 10% FBS, 100 U/ml penicillin, and 100 mg/ml streptomycin. The cells were grown at 37°C and 5% CO2 in humidified incubator (ABI 371, Thermo Fisher Scientific Inc, United States).

#### Determination of NO Production

Nitric oxide (NO) production was measured with the Griess reagent (Sigma-Aldrich, Darmstadt, Germany). Briefly, RAW264.7 cells (1 × 10^5^ cells/well) were incubated in a 48-well plate with different concentrations of test materials for 1 h. Cells were stimulated with lipopolysaccharide (LPS, 1 μg/ml) for 24 h. Supernatants (100 μl) were collected, mixed with an equal volume of the Griess reagent, and incubated at 37°C for 10 min. Afterward, optical density (OD) was measured at 540 nm using a microplate reader (xMark, BIO-RAD, CA, United States). Acetylsalicylic acid (aspirin) (100 µM) was used as positive control in all subsequent assays (Gao et al., 2015). Each experiment was repeated twice, and measurements were taken in triplicate.

#### Measurement of Pro-Inflammatory Cytokine (PGE2, TNF-α, IL-1β, and IL-6) Production

The production of pro-inflammatory cytokine was determined by commercially available ELISA kits (Bio-Rad, CA, United States; Sigma-Aldrich, Darmstadt, Germany). RAW 264.7 cells (1 × 105 cells/well) were plated in 48-well plates and incubated with test material for 1 h prior to LPS (1 μg/ml) stimulation. Cell-free culture supernatants were collected for the determination of PGE2, IL-1β, IL-6, and TNF-α concentration according to the manufacturer’s instructions.

#### RT-qPCR Analysis

Total RNA from RAW 264.7 cells was extracted using TRIzol reagent (Invitrogen, United States) according to the manufacturer’s instructions. First strand complementary DNA (cDNA) was synthesized by using MMLV based reverse transcriptase kit (Invitrogen, United States). Afterward, cDNA was amplified with gene-specific primers using 2Xn-Taq polymerase mixture (Enzynomics, Korea). The primer sequences are listed in Supplementary Table S1.

### Statistical Analysis

Data were analyzed statistically by performing one-way analysis of variance (ANOVA) followed by Duncan’s New Multiple Range Test using SPSS version 21 (Chicago, IL).

## Results

### Comparative Metabolomics of Roots and Aerial Parts of *G. uralensis*

Roots of *G. uralensis* are mostly used in Chinese traditional medicine. Since the aerial parts are normally discarded, we wonder if these parts could also be utilized. In an analysis of the biomass distribution, it was found that roots accounted about 30% of the whole plant biomass (Figure 1). Therefore, medicinal evaluation of the aerial parts must be performed.

**FIGURE 3:**
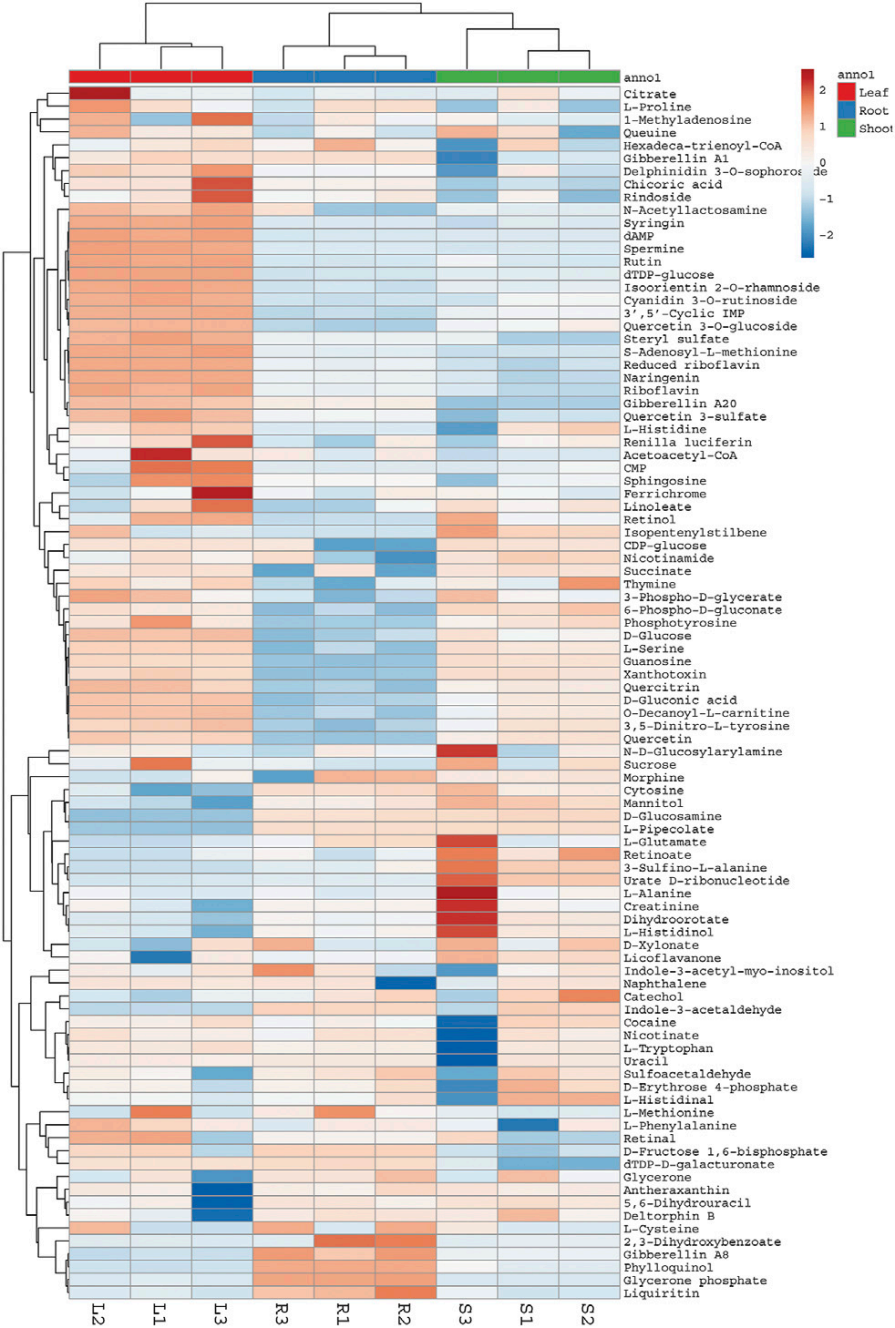
Heat map showing relative abundance of different compounds detected in aerial parts and roots of *G. uralensis*. Heat map was constructed using web based ClustVis tool. L = Leaves, R = Roots, S = Shoots.

Considering the lack of research dealing with the use of the non-traditional (aerial) parts of *G. uralensis* for the exploration of medicinally valuable compounds, a preliminary study was performed focusing on the comparative metabolomics of the roots and aerial parts of this plant. The extracts of aerial and below ground parts of *G. uralensis* were analyzed by GC/MS and UHPLC-ESI-QTof MS/MS analysis (Figure 2). Analysis of the mass spectrum data led to the identification of 79 compounds from the leaves of *G. uralensis*. The identified compounds can be seen in Tables 1 and 2, along with the main identification parameters obtained from the existing databases. Based on our comparative study, both traditional (roots) and non-traditional (aerial) parts showed a varying profile of different compounds like phenolic, saponins, flavonoids, flavonoid glycosides, coumarins, chalcones, and tannins. To provide a global overview, the relative abundance of the compounds in different parts of the plant is shown in a heatmap (Figure 3). The most abundant group of compounds included phenolic acids and their derivatives, which were identified in both positive and negative ionization mode that generated [M + H] and [M-H] precursor ions (Table 1). Another group identified in the leaf samples was the medicinally valued flavanones and glycoside compounds, which are characteristic of *Glycyrrhiza* spp. Other important groups of compounds putatively identified in the leaf samples were saccharides, tannins, and sulfoxides. In addition, lipids and their derivatives were identified in the samples in [M + H] and [M-H] modes. Overall, the results show that the leaves of *G. uralensis* contain a high diversity of all the examined classes of compounds when compared with the roots and shoots (Figure 2).

**TABLE 2:** Comparative quantifications of some major bioactive compounds presented in different parts of *G. uralensis.*

No	Compound name	MS (m/z)	Content (µg ribitol equivalent/g of dry weight)				References
			Roots	Shoots	Leaves		
1	Amentoflavone	539[M + H]+	63.05 ± 03.21	25.38 + 03.82		41.09 + 03.81	Yu et al. (2017)
2	Caffeic acid	181[M + H]+	ND	ND		108.54 + 07.15	Ammar et al. (2017)
3	Feraulic acid	159[M + H]+	06.14 ± 00.98	1.45 + 00.41		26.87 + 03.43	Ammar et al. (2017)
4	Glucuronic acid	193[M-H]−	20 ± 13.25	21.01 + 05.06		83.21 + 01.81	Japan Mass bank
5	Glyasperin C	330[M + H]+	27.54 ± 04.17	ND		78.59 + 06.40	Japan Mass bank
6	Glycyrrhizic acid	826[M + H]+	387.32 ± 36.71	67.8 + 03.98		165.17 + 19.40	Farag et al. (2012)
7	Glycyuralin B	353[M−H]−	ND	89.21 + 11.20		45.23 + 03.19	Farag et al. (2012)
8	Inflacoumarin	321[M-H]−	305.68 ± 05.71	143.21 + 09.61		203.45 + 15.37	Farag et al. (2012)
9	Isolicoflavonol	553[M-H]−	ND	ND		43.25 + 67.27	Zhang and Ye (2009)
10	Isoliquiritin	429[M + H]+	46.2 ± 03.75	13.84 + 01.34		21.54 + 01.63	Zheng et al. (2008)
11	Isoquercitrin	463[M-H]-	151.6 ± 18.26	23.26 + 03.39		57.85 + 02.43	Hefny Gad et al. (2018)
12	Isoviolanthin	577[M-H]−	13.58 ± 01.91	76.25 + 08.51		29.58 + 01.07	Zheng et al. (2008)
13	Kaempferol 7-O-Glycoside	499[M + H]+	124.6 ± 08.63	21.85 + 01.05		58.69 + 04.90	Siracusa et al. (2011)
14	Liquiritigenin	417[M-H]−	36.67 ± 02.54	ND		4.68 + 00.16	Farag et al. (2012)
15	Liquiritin	255[M-H]−	23.5 ± 03.40	06.35 + 91.73		15.21 + 00.37	Farag et al. (2012)
16	Naringenin	273[M + H]+	0.86 ± 00.65	13.25 + 02.59		18.3 + 00.95	Siracusa et al. (2011)
17	*p*-Coumaric acid	163[M-H]−	07.39 ± 00.54	12.86 + 00.52		63.78 + 07.52	Japan Mass bank
18	Quercetin 3-O-Glycoside	463[M-H]−	3.78 ± 00.29	1.26 + 00.28		15.18 + 00.67	Siracusa et al. (2011)
19	Quinic acid	191[M-H]−	18.7 ± 01.57	13.51 + 01.37		37.57 + 02.19	Japan Mass bank
20	Rutin	609[M-H]−	32.75 ± 02.52	25.23 + 03.40		43.67 + 03.51	Japan Mass bank
21	Sinapic acid	225[M + H]+	06.76 ± 00.56	13.98 + 00.61		53.87 + 04.66	Japan Mass bank
22	Licochalcone B	287[M + H]+	11.20 ± 20.15	36.12 + 51.78		221.72 + 61.34	Japan Mass bank

Compounds were quantified by area normalization with Ribitol used as internal standard compound. Values with ± represents standard error.

The root extract contained higher concentrations of most of the medicinally important compounds (Table 2). Moreover, these compounds showed different abundance among roots shoots and leaves. For instance, roots contained approximately ten times more flavanones (13.64% of the total mass extract) compared with the leaves (4.07% of the total mass extract). Similarly, compounds like liquiritin, glycyrrhetic acid, feraulic acid, and isoquercitrin were found abundantly in the roots when compared with the leaves and shoot. Alternatively, some compounds like caffeic acid, glyasperin C, isolicoflavonol, and isolicoflavonol were found in the leaves but not in the shoots. Quantitative data showed that the leaves of *G. uralensis* contained some phenolic acids (sinapic acid and p-coumaric acid), isoviolanthin, and glycyuralin B in higher concentrations in comparison with the roots and shoots. Indeed, phenolic content in 1 g of leaf extract were equivalent to 9.63% of the total mass extract, which is approximately three times lower as compared to the root extracts (2.87% of the total mass extract).

**FIGURE 4:**
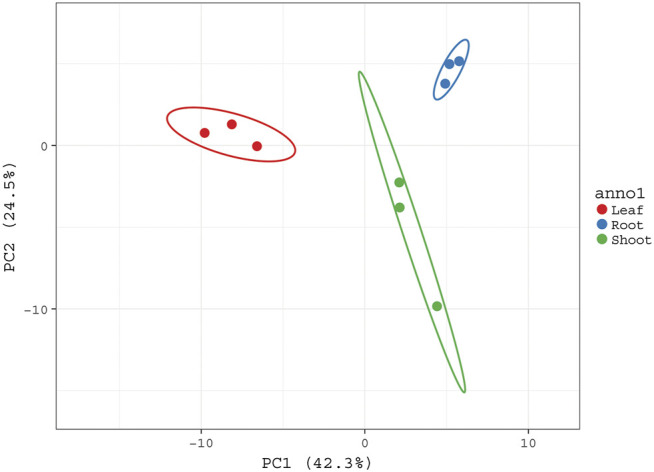
PCA score plot sowing the variability of metabolic profiles of aerial rats and roots of *G. uralensis*. PCA plot was constructed based on abundance of different metabolites present in aerial and roots of *G. uralensis*. All three groups corresponding to different plant parts are well separated from each other based on the variability of their metabolic profiles.

Furthermore, a quantitative data set was created to compare metabolic profile of different plant parts by performing PCA analysis. It showed great extent of variability in the chemical composition of extracts obtained from the roots, shoots, and leaves of *G. uralensis*. PCA plot showed three distinct groups corresponding to different plant parts (Figure 4).

### Isolation and Identification of Anti-Inflammatory Compounds From the Leaves of *G. uralensis*

#### Effect of *G. uralensis* Leaf Extracts on LPS-induced NO Production

**FIGURE 5:**
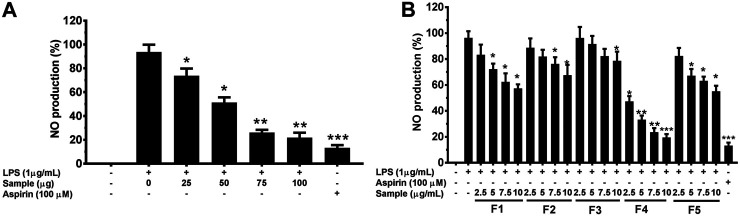
Effects of crude extracts **(A)** and fractions **(B)** EtOH leaf extracts of *G. uralensis* on nitric oxide in RAW 264.7 cells. Cells (1.0 × 105 cells/ml) were stimulated by LPS (1 μg/ml) for 24 h in the presence of rude extracts and fractions at varying concentrations. Culture media were collected in order to measure NO by the Griess reaction. Values are the mean ± standard error of triplicate experiments. *p < 0.05, **p < 0.01 and **p < 0.001 for the comparison with the LPS-stimulated group.

To determine the anti-inflammatory effects of *G. uralensis* leaf extracts, we initially investigated the inhibitory effects of crude extracts (at concentration of 25,50,75 and 100 μg/ml) and fractionated leaf extracts (at concentration of 2.5,5.7.5 and 10 μg/ml) against NO production using LPS-induced RAW 264.7 cells. Among the tested fractions, F4 showed the highest inhibitory effect against NO release (Figure 5).

#### Identification of the Active Compounds and Their Inhibitory Effect in LPS-Induced NO and PGE2 Production

**FIGURE 6:**
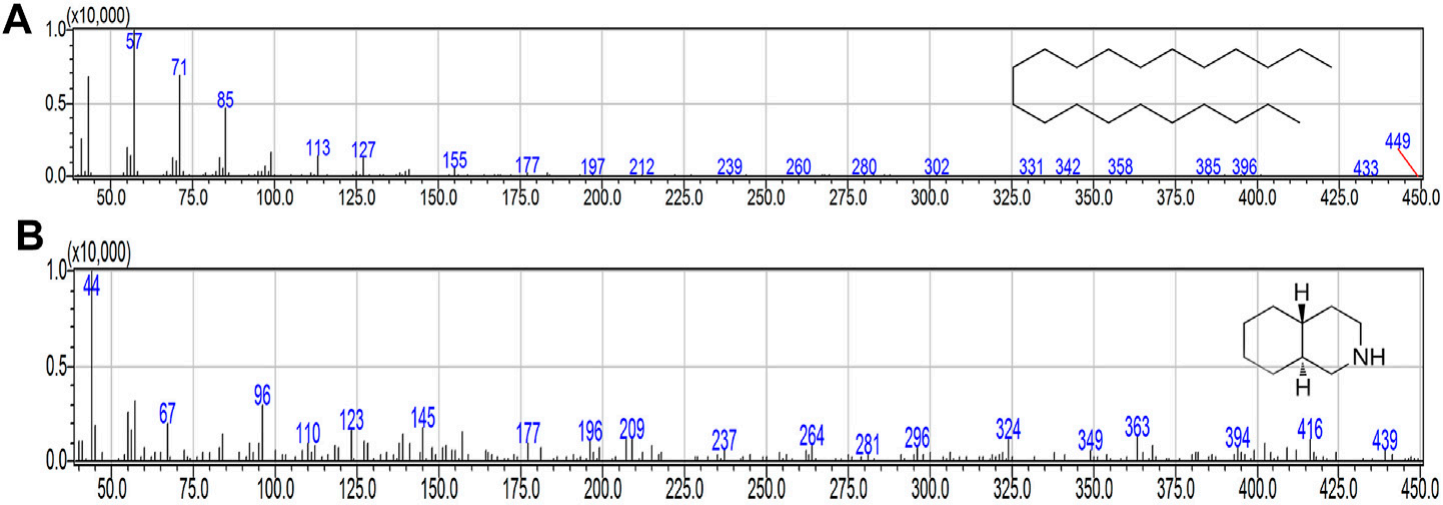
MS spectra of purified compounds. **(A)** henicosane. **(B)** decahydroisoquinoline.

Firstly, the compounds were putatively identified with MS analysis (Figure 6). Afterward, the identification was confirmed by comparing retention and molecular indices with pure authentic internal standards. Compounds belonging to fraction four, were identified as henicosane (1) and decahydroisoquinoline (2) also known as perhydroisoquinoline.

**FIGURE 7:**
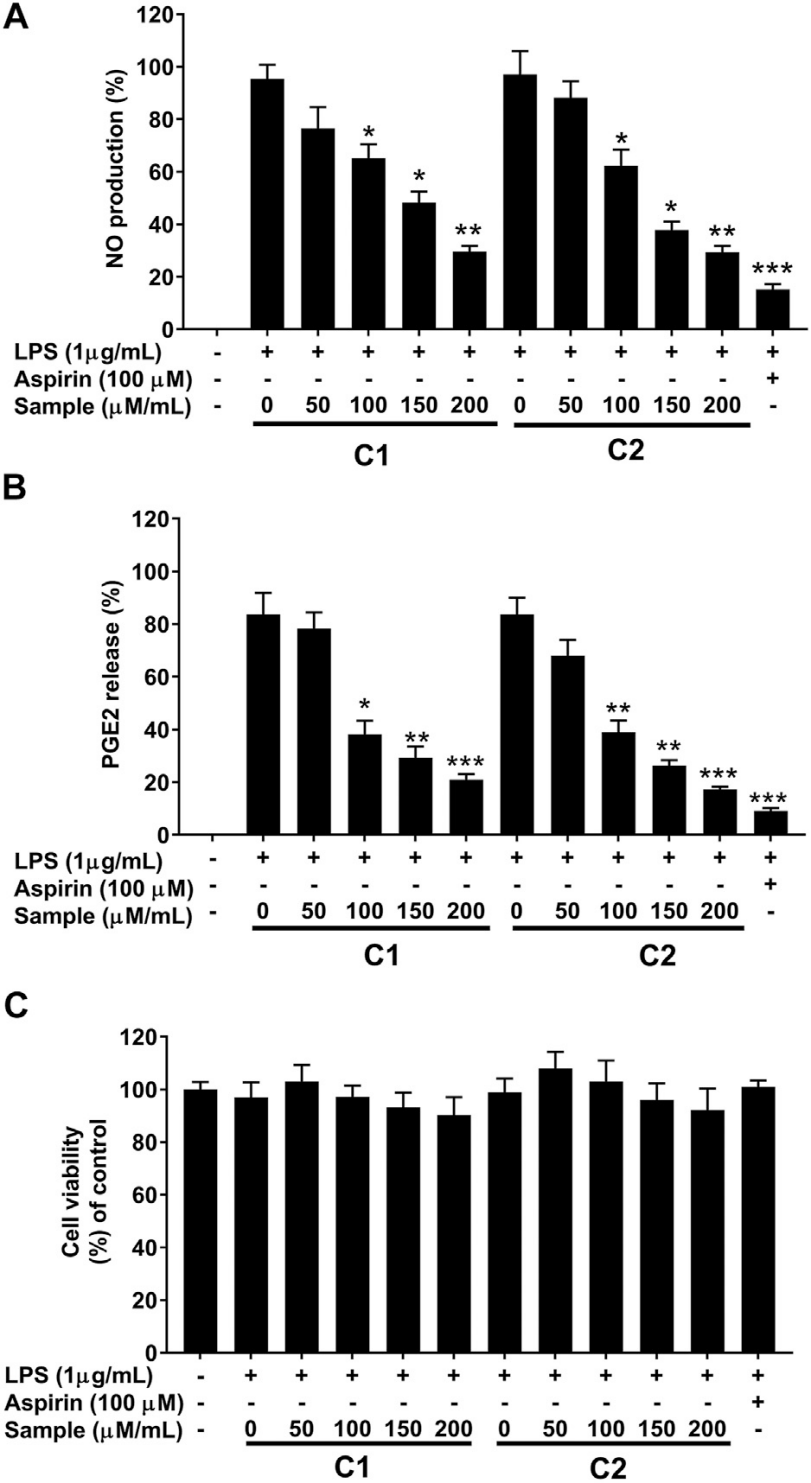
Effects of purified compounds on production of nitric oxide and prostaglandin E2 and cytotoxicity in RAW 264.7 cells. Cells (1.0 × 105 cells/ml) were stimulated by LPS (1 μg/ml) for 24 h in the presence of compounds (50, 100, 150, and 200 µ/ml). Culture media were collected in order to measure **(A)** NO and **(B)** PGE2 production by the Griess reaction and ELISA assay, respectively. **(C)** Cytotoxicity was determined using the MTT method. Values are the mean ± standard error of triplicate experiments. *p < 0.05, **p < 0.01 and **p < 0.001 for the comparison with the LPS-stimulated group.

To assess the inhibitory effect of purified compound 1 and 2 in LPS stimulated RAW 264.7 cells, the production of NO was measured by the Griess reaction and PGE2 by ELISA, respectively. As shown in Figures 7A,B the production of NO and PGE2 was markedly increased by stimulation with LPS. In contrast, treatment with compound 1 and 2 inhibited the production of both mediators in a concentration-dependent manner (Figures 7A,B). This remarkable effect was not related to the nonspecific cytotoxicity, since both compounds showed non-significant effects on RAW 264.7 cell viability, as determined by the methyltetrazolium (MTT) assay (Figure 7C). Hence, the inhibition of NO and PGE2 was due to a direct inhibitory effect of the test compounds.

#### Effect of Purified Compounds in LPS-induced iNOS and COX-2 Expression

**FIGURE 8:**
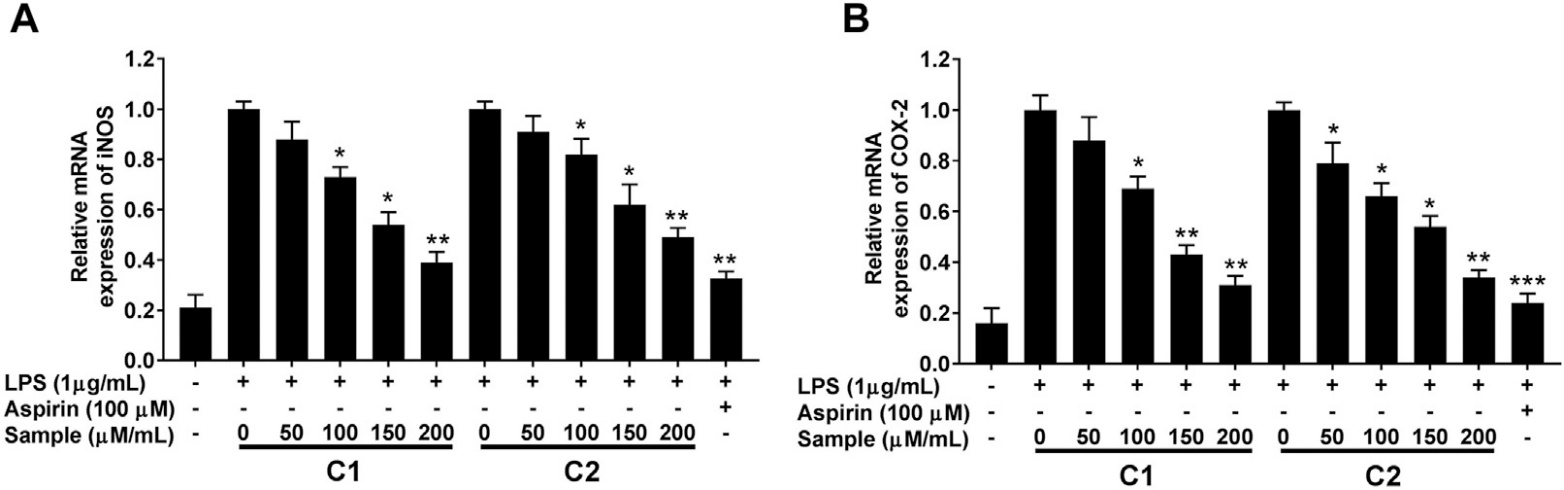
Effects of purified compounds on iNOS **(A)** and COX-2 **(B)** genes expression in RAW264.7 cells. Cells were pretreated with the indicated concentrations of purified compounds for 30 min and simulated with LPS (1 μg/ml) for 24 h. Gene expression was analyzed by quantitative RT-PCR analysis using gene specific primers in a concentration-dependent manner. Values are the mean ± standard error of triplicate experiments. Values are the mean + S.E. of triplicate experiments. *p < 0.05, **p < 0.01 and ***p < 0.001 for the comparison with the LPS-stimulated group. iNOS; nitric oxide synthase, COX-2; cyclooxygenase-2.

Furthermore, the anti-inflammatory effect of the purified compounds was correlated with the expression levels of inducible nitric oxide synthase (iNOS) and cyclooxygenase-2 (COX-2), as revealed by RT-qPCR analysis. LPS simulation significantly increased the expression of these inflammation related genes (Figure 8), whereas, the presence of compound 1 and 2 significantly attenuated their induction in a concentration-dependent manner (Figure 8).

#### Effect of Purified Compounds on LPS-Induced Pro-inflammatory Cytokines Production

**FIGURE 9:**
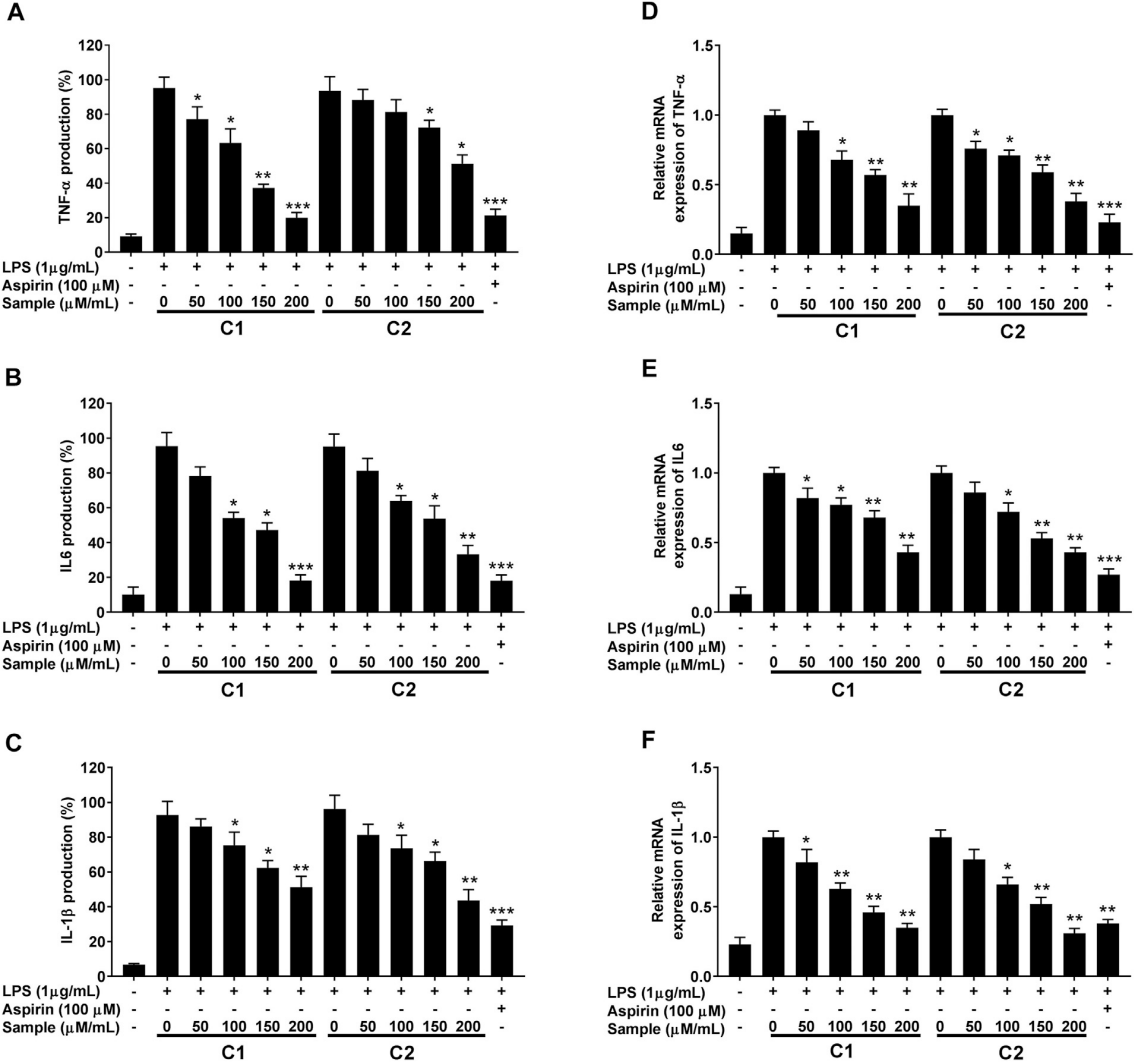
Inhibitory effect of purified compounds on pro-inflammatory cytokine production in RAW 264.7 cells. Cells (1.0 × 105 cells/ml) were stimulated by LPS (1 μg/ml) for 24 h in the presence of compounds (2.5, 5, and 10 µ/ml). Supernatants were collected, the TNF-α, IL-6 and IL-1β production in the supernatants was determined by ELISA **(A–C)**. Gene expression was analyzed by quantitative RT-PCR analysis using gene specific primers **(D–F**). Values are the mean ± standard error of triplicate experiments. Values are the mean ± standard error of triplicate experiments. *p < 0.05, **p < 0.01 and ***p < 0.001 for the comparison with the LPS-stimulated group.

The inhibitory effect of purified compounds on LPS-simulated RAW 264.7 cell was further analyzed by measuring the changes in the release and the transcription levels of pro-inflammatory cytokines (IL-1β, IL-6, and TNF-α) using ELISA and RT-qPCR analysis, respectively. As can be seen in Figure 9, treatment with compound 1 and 2 lowered the expression levels of all tested LPS-induced pro-inflammatory cytokines at both mRNA and protein levels in a concentration-dependent manner.

## Discussion

*Glycyrrhiza* spp. plays an important role in many prescriptions used in complementary and alternative medicines (Ayeka et al., 2017). These plants are used in traditional Chinese medicine to treat many diseases and act as ingredients in the confectionary industry in Japan. To obtain a chemical profile of the roots and aerial parts of *G. uralensis*, an analytical method based on GC/MS and UHPLC/MS was developed. Simultaneously acquired UHPLC/MS total ion chromatograms for the roots, shoots, and leaves extracts of *G. uralensis* are show in Figure 2; while the identities, retention times, and observed molecular and fragment ions for metabolites are presented in Tables 1 and 2. Metabolites were identified by matching their m/z attributes with those reported in the literature, standard compound libraries (NIST and Wiley), and the “Japan Mass Bank” (Horai et al., 2010). When possible, the identification was confirmed with standard compounds available in-house.

In this study, the overall chemical profile of *G. uralensis* in terms of the types and contents is in agreement to previous studies (Bai et al., 2020; Kitagawa et al., 1993; Song et al., 2017; Yu et al., 2021). The roots and aerial parts showed the presence of varying abundance of different classes of phytochemicals such as total phenolics, flavonoids, tannins, and saccharides. As shown in Table 2, some of these medicinally valuable compounds were quantified by the normalization of peak areas with authentic internal standards. Remarkably, more than 40 phenolic compounds were identified in the leaves of *G. uralensis*, suggesting that their abundance is related to the medicinal usefulness of the aerial parts when compared with the roots (Abureidah et al., 2014). Consistently, the UHPLC/ESI/MS total ion chromatogram of the leaf extract of *G. uralensis* showed the presence of several medicinally valuable phenolic acids: sinapic acid with m/z [M + H]+ of 225, p-coumaric acid with m/z [M + H]+ of 163, and ferulic acid with m/z [M + H]+ of 159; as well as flavonoids: isoliquiritin showing m/z [M + H]+ at 429 and isoquercitrin with m/z [M + H]+ at 463 (Table 1). Some previous studies have also reported presence of same types of medicinally important flavonoids (Fukai et al., 1991; Yuldashev 1998) and phenolics (Nomura et al., 2002) in aerial and belowground parts of *G. uralensis*.

Moreover, O- and C-glycosylated forms were also identified from the aerial parts of *G. uralensis*. The C-glucosides entities were proposed based on their fragmentation pattern, which involved the sugar moiety by losses of 2, 3, or 4 (HCHO) (De et al., 2012). However, further stereochemical differentiation of the isomers was not possible by UHPLC/ESI/QTof/MS [20]. Some dominant glycosides in the aerial parts and roots of the plant were: kaempferol 7-O-glucoside characterized by its main fragment at m/z [M + H]+ 499 (relative intensity 100%); isoorientin 2-O-rhamnoside with m/z [M + H]+ at 595; and quercetin 3-O-glycoside, with a main fragment at m/z [M + H]+ 463 (relative intensity 100%). The concentration of quercetin 3-O-glycoside was higher in the leaves than in the roots, whereas the opposite was seen for kaempferol 7-O-glucoside that was present in higher quantities in the root extracts (Table 1). The same types of glycosides have been reported in aerial parts of *G. uralensis* (Jia et al., 1992).

Beside polyphenolic compounds, other polar compounds were identified including sugars, amino acids, and organic acids. Monosaccharides were detected at m/z 195 (galactose), m/z 259 (glucose 6-phosphate), and m/z 341 (fructose 1,6-bisphosphate). Amino acids eluted between 1 and 14 min corresponding to alanine (m/z 90), proline (m/z 115), and tryptophan (m/z 205). The known organic acids were identified as sinapic acid, stearic acid, usnic acid, and cholic acid, among others (Table 1).

The PCA was performed to highlight the varying metabolic profiles of the aerial and underground parts of *G. uralensis*. Pre-processed metabolomics data sets from different plant parts were analyzed to generate a PCA plot in which three different groups could be discriminated, thus indicating the varying distribution of components in the leaves, roots, and shoots of *G. uralensis* (Figure 4). In our study, the integration of data obtained from GC/MS and UHPLC/QTof/MS into a single matrix for PCA allowed the clear separation of extracts from the different plant parts, thereby highlighting the importance of both techniques for sample classification.

In our effort to screen bioactive compounds from the aerial parts of *G. uralensis*, a bio-guided fractioning allowed the isolation of two phytochemicals (henicosane-1 and decahydroisoquinoline-2) that exhibited significant anti-inflammatory effects. Furthermore, the mechanisms mediating this effect were investigated using RAW 264.7 cells. The results revealed that both the compounds significantly reduced the production of NO and PGE2, as well as the mRNA levels of iNOS and COX-2 in LPS-stimulated RAW 264.7 macrophages (Figures 5, 7).

Henicosane belongs to alkanes that are found in various eukaryotic organisms (Coates et al., 2014). These compounds are considered among the main constituents in the most of the plants (Mathis and Ourisson, 1964). Long chain alkanes have been widely isolated from plant fractions possessing medicinal properties (Aiello et al., 2000; Bush and McInerney 2013; Ghasemi Pirbalouti et al., 2014). Methane, a simplest alkane has shown the protective effect to inhibit some inflammatory signals caused by LPS in macrophages and suppress immune response in mice by intensifying IL-10 expression through PI3K/AKT/GSK-3β pathway (Zhang et al., 2016). Second bioactive compound (decahydroisoquinoline) purified in study is an isoquinoline alkaloid. The phytochemical and biological investigation of different plants have led to the isolation of several isoquinoline alkaloids with medicinal properties (Iranshahy et al., 2014; Khan and Kumar 2015; Haider et al., 2018; Bala et al., 2019). An increasing number of recent studies have reported that alkaloids are effective for treating inflammatory disorders and bring good ground for hope of drug development (Peng et al., 2019).

Macrophages play a key role in the immunopathological phenomena during inflammation and infection owing to their phagocytic and cytotoxic capacities (Laskin and Pendino 1995; Mosser and Edwards 2008; Decano et al., 2016). Pro-inflammatory mediators (NO and PGE2) and cytokines (IL-1β, IL-6, and TNF-α) are overproduced by macrophages under inflammation (Fujiwara and Kobayashi 2005; Jin et al., 2008). Lipopolysaccharides (LPS)s are the main components of the cell wall of Gram-negative bacteria, which upon recognition by murine macrophages, elicit their activation with a distinctive up-regulation of iNOS expression (Nathan and Xie 1994). In fact, high levels of NO production are of crucial importance in the process of macrophage response (MacMicking et al., 1997). Therefore, the suppression of NO is considered an important therapeutic target to treat inflammation (Batkhuu et al., 2002; Lee et al., 2008; Yoshitake et al., 2008). In our study, we successfully established that the compounds C1 and C2 isolated from the leaves of *G. uralensis* are strong suppressors of NO production by LPS-stimulated RAW 264.7 murine macrophages. This was accompanied by the inhibition of PGE2 and inflammatory cytokines (IL-1β, IL-6, and TNF-α), as assessed by ELISA and qRT-PCR analysis (Figures 8, 9). As both test compounds showed an effect in all the evaluated downstream targets, including iNOS and COX-2 enzymes, our findings suggest that the anti-inflammatory effect of compound 1 and 2 from *G. uralensis*, may be due to the targeting of upstream signaling such as that related to the mitogen-activated protein (MAP) kinase or the nuclear factor (NF)-κB signal pathways.

## Conclusion

Our findings suggest that henicosane 1) and decahydroisoquinoline 2) isolated from the leaves of *G. uralensis* are valuable anti-inflammatory metabolites. This study supports the pharmacological importance of the non-traditional aerial parts of *G. uralensis* as potential sources of new natural compounds for the treatment of inflammation. Secondly, these aerial parts, which are currently considered an agro-industrial waste, can be used to recover liquiritin and some other medicinally valuable components.

## Data Availability

The datasets presented in this study can be found in online repositories. The names of the repository/repositories and accession number(s) can be found in the article/Supplementary Material.
