# Supplementary material for: Metabolomic and Pharmacologic Insights of Aerial and Underground Parts of Glycyrrhiza uralensis Fisch. ex DC. for Maximum Utilization of Medicinal Resources
Source: Front Pharmacol. 2021 Jun 1;12:658670. doi: 10.3389/fphar.2021.658670 (PMC8204184; doi:10.3389/fphar.2021.658670)
Supplement: Supplementary file 1 [file DataSheet1.PDF]

**Supplementary table 1.** The primer sequences used in this study.

| Gene | Sequences (5'-3') |
|---|---|
| iNOS | GACAAGCTGCATGTGACATC<br>GCTGGTAGGTTCCTGTTGTT |
| COX-2 | TCCAGATCACATTTGATTGA<br>TCTTTGACTGTGGGAGGATA |
| IL-1β | GATCCACACTCTCCAGCTGCA<br>CAACCAACAAGTGATATTCTCCATG |
| IL-6 | TTCCATCCAGTTGCCTTCTTG<br>GGGAGTGGTATCCTCTGTGAAGTC |
| TNF-α | ATGAGCACAGAAAGCATGAT<br>(TACAGGCTTGTCACTCGAAT |
| β-actin | ATCCTGCGTCTGGACCTGGCT<br>CTGATCCACATCTGCTGGAAG |

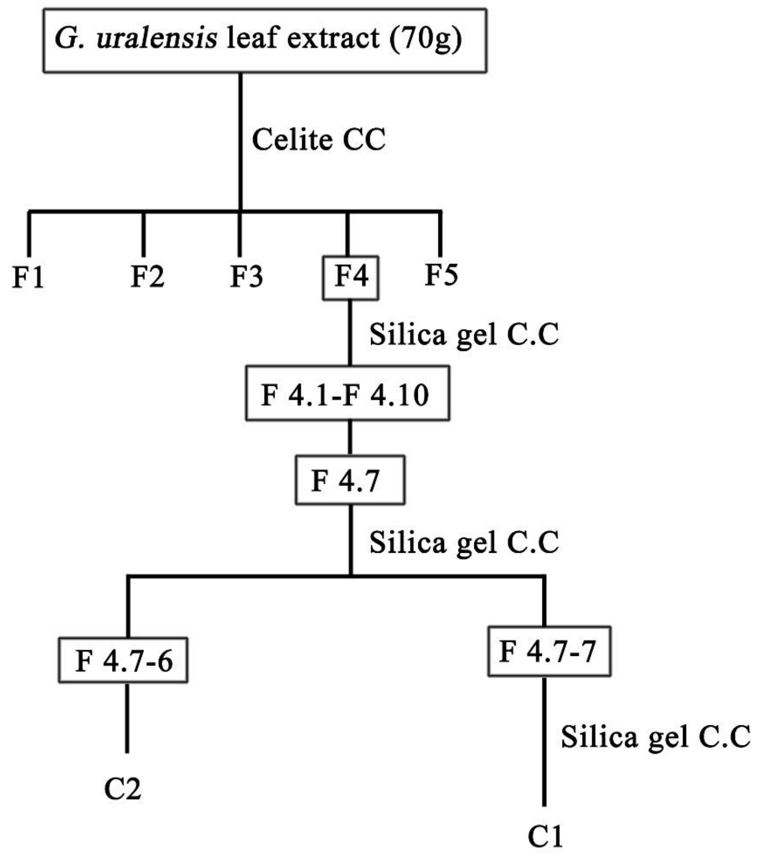


**Supplementary figure 1.** Isolation scheme of the isolated compounds from leaf extracts of *G. uralensis.*
